# Supplementary material for: Guanosine-Mediated Anxiolytic-Like Effect: Interplay with Adenosine A1 and A2A Receptors
Source: Int J Mol Sci. 2020 Dec 5;21(23):9281. doi: 10.3390/ijms21239281 (PMC7729560; doi:10.3390/ijms21239281)
Supplement: Supplementary file 1 [file ijms-21-09281-s001.pdf]

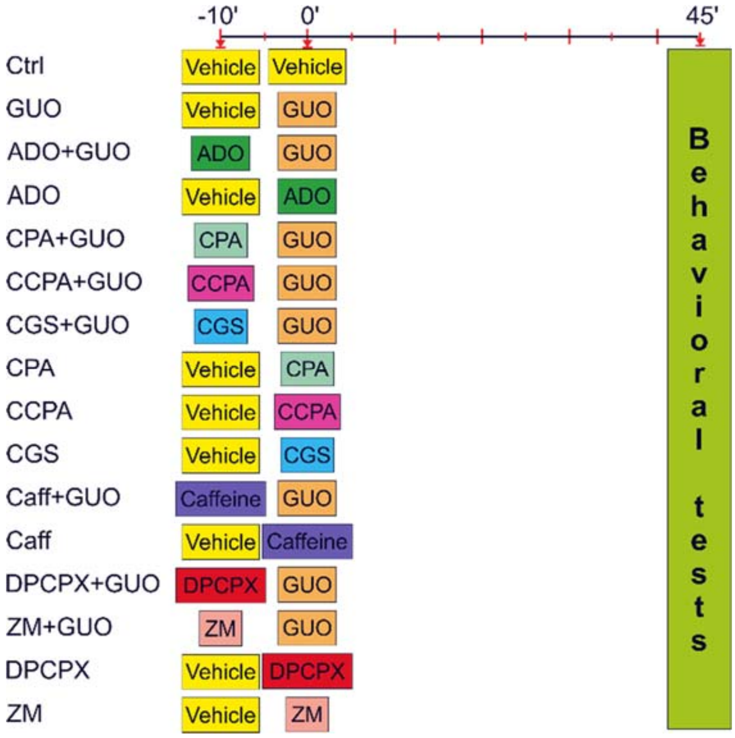

Figure S1. Scheme of drug treatments and behavioral experimental design

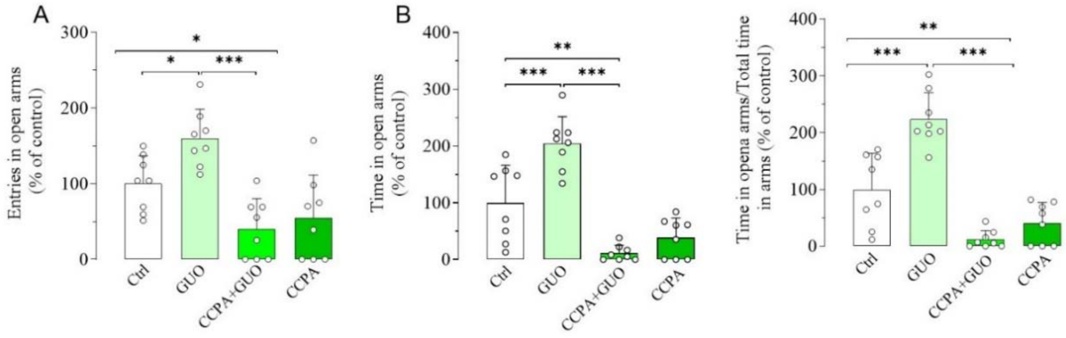

Figure S2. Functional competition between GUO and A1R selective agonist CCPA on GUO-mediated anxiolytic-like effect evaluated by EPM test during a 5 min session. Pretreatment with CCPA (0.1 mg/kg) was able to completely block the anxiolytic-like effect of GUO (30 mg/kg) with significant decrease in (**A**) number of open arm entries [$F(3,28)=12.24$, $p < 0.0001$], (**B**) time spent in open arms [$F(3,28)=28.83$, $P<0.0001$] and (**C**) the ratio of time in open arm and total time in arms [$F(3,28)=36.12$, $P<0.0001$], when compared to GUO-treated and control (Ctrl) group. Each bar represents the mean value ± SD. Tukey test: * $p < 0.05$, ** $p < 0.01$, *** $p < 0.001$. Ctrl raw mean values: (**A**) 2.75; (**B**) 122.25 s; (**C**) 0.46.

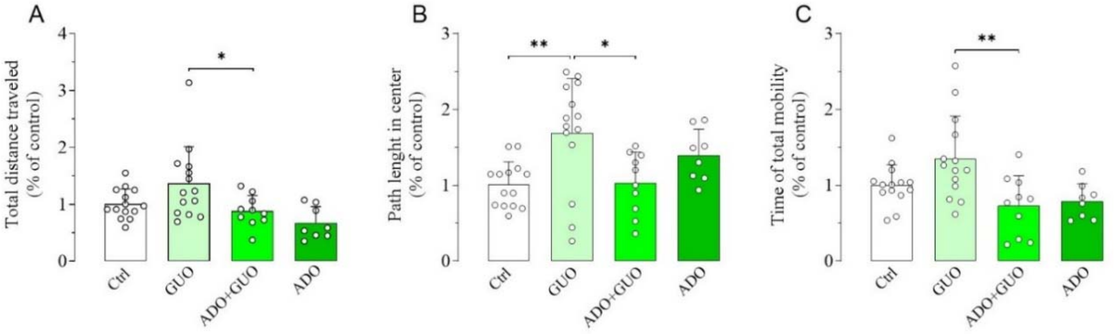

Figure S3. Effects of GUO on locomotor activity evaluated by OFT test during a 5 min session. (**A–C**) The OFT data analysis revealed that GUO treatment (30 mg/kg) significantly increased the path length in center, as compared to control (Ctrl) group. Pretreatment with nonselective ARs agonist ADO significantly decreased the total distance travelled, the path length in center and the time of total mobility, as compared to GUO-treated group. ADO treatment alone did not affect behavior as compared to control group. Total distance traveled [$F_{(3,42)}$=5.34, $p < 0.005$], path length in center [$F_{(3,42)}$ =5.605, $p <$ 0.005], time of total mobility [$F_{(3,42)}$ =5.844, $p < 0.005$]. Each bar represents the mean value ± SD. Tukey test: *$P < 0.05$, **$P < 0.01$. Ctrl raw mean values: (**A**) 3.47 m; (**B**) 0.6 m; (**C**) 50.05 s.
